# Age-Specific Differences in Foreign Bodies Ingested by Children: A Cohort Study of 252 Japanese Cases

**DOI:** 10.3390/medicina56010039

**Published:** 2020-01-19

**Authors:** Jumpei Fujisawa, Tomokazu Mutoh, Kengo Kawamura, Ryuta Yonezawa, Maiko Hirai, Ichiro Morioka

**Affiliations:** Department of Pediatrics and Child Health, Nihon University School of Medicine, 30-1, Oyaguchi, Kami-cho, Itabashi-ku, Tokyo 173-8610, Japan; fujisawa.junpei@nihon-u.ac.jp (J.F.); mutoh.tomokazu@nihon-u.ac.jp (T.M.); kawamura.kengo@nihon-u.ac.jp (K.K.); yonezawa.ryuta@ims.gr.jp (R.Y.); hirai.maiko@nihon-u.ac.jp (M.H.)

**Keywords:** age, children, cohort study, foreign-body ingestion

## Abstract

*Background and Objectives:* When children accidentally ingest foreign bodies, they may be unable to communicate adequately; it is often difficult to identify the causative foreign body unless someone is watching over them. In such instances, to identify the causative foreign body during clinical practice, we aimed to determine if it varies according to age. *Materials and Methods:* From April 2013 to June 2018, 252 records of pediatric patients with a confirmed diagnosis of foreign-body ingestion were retrospectively examined in a Japanese university hospital. Comparisons among multiple age groups, according to type of ingested foreign body, were analyzed using Kruskal‒Wallis tests. The differences between the individual data were tested using the Steel‒Dwass test. *Results:* The median age of the patients was 15 months, and of the total patients, 140 were boys (55.5%). The types of foreign bodies ingested were as follows, in order of frequency: cigarettes (*n* = 44, 17%, median age: 12 months), plastics (*n* = 43, 17%, median age: 11 months), chemicals (*n* = 27, 11%, median age: 13 months), internal medicines (*n* = 26, 10%, median age: 33 months), and metals (*n* = 26, 10%, median age: 35 months). The median age was significantly different among the types of causative foreign bodies (*p* < 0.01). The patient age for the ingestion of cigarettes was significantly younger than that for ingesting metals or coins. The age for ingesting internal medicines was significantly older than that for ingesting plastics, cigarettes, paper, or chemicals (*p* < 0.01). *Conclusions:* The causative foreign body ingested differed according to age. This will be valuable information for physicians that encounter pediatric patients who may have ingested an unknown foreign body in Japanese pediatric emergency or general practice settings.

## 1. Introduction

Foreign-body ingestion is a common encounter during pediatric emergencies or in general practice; importantly, however, it may lead to mortality [1,2,3,4,5]. The 2016 American Association of Poison Control Centers’ National Poison Data System reported 1,810,030 incidents of foreign-body ingestion in a year [2]. Eighty percent of foreign-body ingestion had occurred in infants, toddlers, and children; it had occurred most commonly from the ages of 6 months to 3 years [3,6,7,8,9,10]. Foreign-body ingestion in children is different from adults―over 90% of cases of foreign-body ingestion in children are accidental, and few cases involving psychiatric illness are observed in children [8,11,12,13,14]. Early detection is crucial as emergency first aid is sometimes required for patients.

The ingestion of foreign bodies by young children may be observed by parents or other guardians; however, around 40% of incidents are not witnessed [3,15]. Without a witness, it can be difficult to identify the causative foreign body, as children may be unable to communicate sufficiently and the exhibited symptoms are not typically observed [3,16]. In such cases, it may be important for pediatricians and acute-care physicians to identify the causative foreign body. Additionally, considering the recent lifestyle changes of people in Japan, an investigation of causative foreign bodies is warranted. The aim of this study was to determine differences in the causative foreign bodies ingested by juvenile patients according to age.

## 2. Materials and Methods

### 2.1. Setting

We conducted a single-center cohort study at Nihon University Itabashi Hospital, north of Tokyo, Japan. This center provides medical services that include emergency medicine for pediatric patients 24 h/day, 365 days per year. Approximately 39,000 pediatric patients per year visit the hospital, and 1800 pediatric patients per year are hospitalized. Around 40 pediatricians work at the hospital on a full-time basis.

### 2.2. Study Design and Methods

A retrospective observation study was conducted. From April 2013 to June 2018, there were 252 patients under 16 years old with a confirmed diagnosis of foreign-body ingestion in the pediatric emergency or general pediatrics departments. The background characteristics, including age, sex, place of occurrence, provision of first aid, symptoms at the time of ingestion, month when the foreign-body ingestion occurred, and types of foreign bodies ingested, were analyzed. Following this, the relationship between the foreign bodies and the patients’ ages was examined. This study was approved by the clinical-research ethics review board of Nihon University Itabashi Hospital (ethical code number: RK-181211-05; date of approval: 8 March 2019). Formal, written informed consent was not required, owing to the data having originated from our regular practices and the retrospective nature of the study. In light of this, we opened this study project to the public using our website. The study procedures were in accordance with the ethical standards of the responsible committee on human studies and with the Helsinki Declaration of 1975, as revised in 2008.

### 2.3. Definition

For this retrospective study, the patients were selected from medical records according to the International Classification of Diseases, Tenth Edition, for foreign-body ingestion (code numbers: T17.2, T17.3, and T18.1–9), which include a foreign body in the pharynx, larynx, mouth, esophagus, stomach, small intestine, colon, anus and rectum, and other parts of the alimentary tract.

### 2.4. Classification

The foreign bodies ingested were divided into 13 groups by the frequency of their ingestion by children: cigarettes (e.g., cigarettes, electronic cigarettes, and water filled with cigarette butts), plastics (e.g., plastic bags, film packages, and plastic toys), internal medicines (e.g., prescribed medicines for parents and prescribed medicines for older brothers or sisters), chemicals (e.g., detergents, cosmetics, and air fresheners), metals (e.g., Japanese pinball balls, bolts and nuts, and metallic toy parts), batteries (e.g., coin batteries), coins, paper (e.g., tissues and stickers), magnets, rubber (e.g., hair elastics, rubber bands, and rubber handles), glass (e.g., marbles and rhinestones), food and drink (e.g., candies, chunks of meat, and alcohol), and others.

### 2.5. Statistical Analysis

Statistical analyses were performed using JMP version 14 (SAS Institute Japan, Tokyo, Japan). The average number of patients per month was analyzed using Tukey‒Kramer’s honestly significant difference test. Comparisons among multiple median ages and ingested foreign bodies were conducted using the Kruskal‒Wallis test. Differences between individual data were tested using the Steel‒Dwass method. Age-specific differences were determined according to the following categorization: children <12 months of age (i.e., infants), children aged 1–6 years (i.e., toddlers and preschoolers), and children ≥7 years of age (i.e., school-age children). Comparisons between groups were performed using Pearson’s chi-square test for m x n tables.

## 3. Results

### 3.1. Patient Characteristics

**Figure 1:**
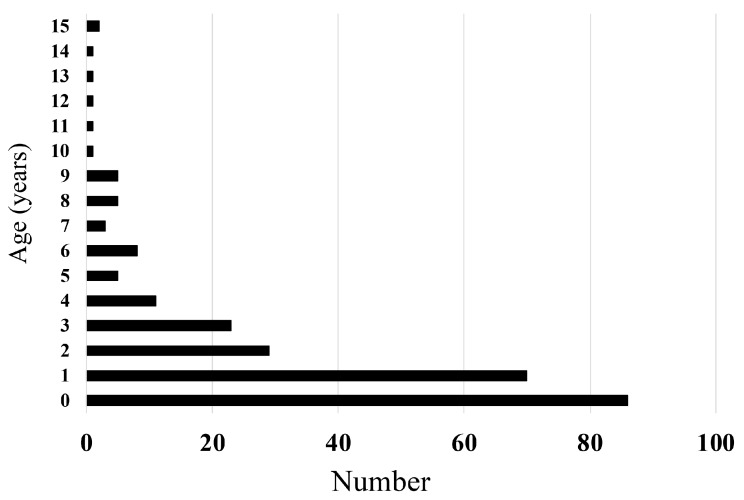
Age at ingestion of the foreign body.

**Figure 2:**
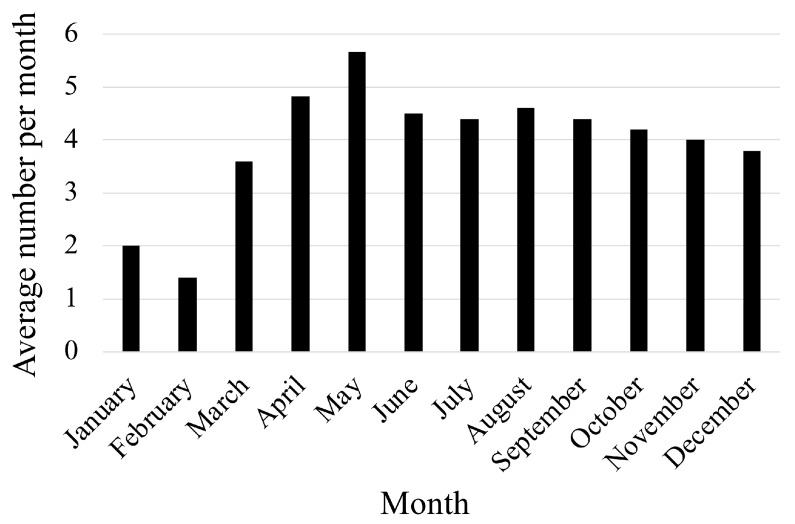
Average number of patients who ingested foreign bodies, classified by month.

The median age of all patients with a foreign-body ingestion was 1 year and 3 months. Infants aged <12 months ingested foreign bodies more frequently; the number of cases of foreign-object ingestion decreased as age increased. The recurrence of foreign-body ingestion was calculated at 2%. The most frequent location of ingestion was at home. Sixty-seven patients were provided with first aid by bystanders, such as parents. The first aid included water provision, extraction of objects from the mouth, and inducing vomiting (Table 1 and Figure 1). For 60% of patients, there were no symptoms. In the remaining 40% of patients, symptoms, such as nausea and vomiting, coughing, and the disturbance of consciousness (Table 1), were recorded. The number of patients tended to be less in January and February with more cases in April and May (mean: 1.4/month in February and 5.7/month in May, *p* = 0.089, Figure 2).

### 3.2. Causes

**Figure 3:**
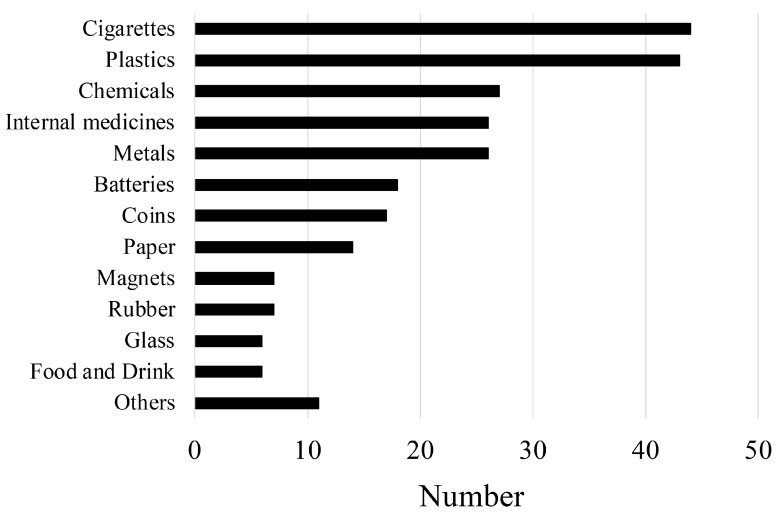
Types of foreign bodies ingested according to frequency.

The types of foreign bodies ingested are shown in Figure 3. The major types of foreign bodies ingested were cigarettes (*n* = 44, 17%), plastics (*n* = 43, 17%), chemicals (*n* = 27, 11%), internal medicines (*n* = 26, 10%), and metals (*n* = 26, 10%). There were no differences in the types of ingested foreign bodies between male and female patients (*p* = 0.678).

### 3.3. Age-Specific Characteristics and Types of Foreign Bodies

When classified by age, i.e., infants, toddlers and preschoolers, and school-age children, the age-specific background characteristics of sex, presence of symptoms, occurrence at home, and first aid provision were not statistically significant. However, the major causative foreign bodies were significantly different according to age (*p* < 0.01, Table 2). When divided into male and female patients, the major causative foreign bodies were also significantly different according to age, both in males (*p* < 0.01) and females (*p* < 0.01).

**Figure 4:**
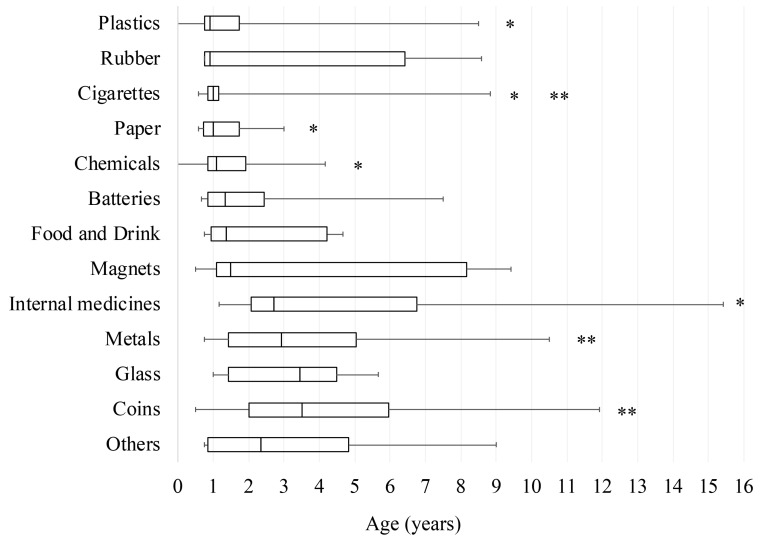
Comparison of patient age at the ingestion of each foreign body. Data are shown as the minimum, lower quartile, median, upper quartile, and maximum as box-and-whisker plots. *p* < 0.01 for * internal medicines vs. plastics, cigarettes, paper, or chemicals, ** cigarettes vs. metals or coins.

The patient age for the type of foreign body ingested is shown in Figure 4. Significant differences were found according to the age at which different causative foreign bodies were ingested (*p* < 0.01). The patient age for the ingestion of cigarettes (median, 12 months) was significantly younger than that for ingesting metals (median, 35 months) or coins (median, 42 months). The age for ingesting internal medicines (median, 33 months) was significantly older than that for ingesting plastics (median, 11 months), cigarettes (median, 12 months), paper (median, 12 months), or chemicals (median, 13 months) (*p* < 0.01). Three (50%) from a total of six school-age patients who ingested internal medicines had an intentional overdose, and all three of these patients had a medical history of psychological or psychiatric disorders.

## 4. Discussion

Pediatric patients who ingested a foreign body were retrospectively studied in a Japanese university hospital. Although there are clinical reports regarding treatments, such as endoscopy, and the involvement of the various parts of the alimentary tract (e.g., foreign bodies in the esophagus or stomach) [3,6,7,8,9,10], there are few Japanese cohort studies regarding the clinical background of patients presenting with foreign-body ingestion. Considering this, the current study was performed to identify age-specific differences in the ingestion of foreign bodies by children. As a result, age-specific differences were found in the types of ingested foreign bodies. The reason is that what children get hold of depends on their age. As infants cannot distinguish between what they do and do not eat, whatever they pick up is put in their mouth. In school-age children, foreign bodies are ingested accidentally.

Among pediatric patients, infants ingested foreign bodies most frequently, but the frequency of ingestion events decreased as age increased. This result is consistent with previous reports from non-Japanese countries [3]. The major type of foreign-body ingestion was cigarettes, although the percentage of smokers in Japan has been decreasing (24.1% in 2007 to 17.7% in 2017) [17]. Plastic products, such as bags and film packages, and chemicals, such as detergents and cosmetics, were also frequently ingested; all are easily accessible to children in typical Japanese homes. As most infant cases occur at home, keeping objects out of reach of infants and toddlers should be prioritized.

Guardians can easily envision that cigarettes, internal medicines, and metals are toxic objects, and thus, such objects are more likely to be kept out of reach. However, the toxicity of plastics may not be an obvious consideration; therefore, close attention should be paid to children who have access to plastic products. Coins are a major type of ingested foreign body in Western countries [18] but were not so in our Japanese cohort. Due to changing technological lifestyles, a shift from the use of coins to electronic transactions is becoming typical in many countries. Cases of coin ingestion may, as a result, decrease more in the future.

Forty percent of the patients had symptoms at the time of ingestion in this study. A previous study reported that 54% of 325 pediatric patients had transient symptoms [18], which is similar to our result. As there are no apparent specific symptoms associated with different foreign bodies, it may often be difficult to diagnose according to the symptoms alone. Although the most frequent place where ingestions occurred was at home, preschooler accidental ingestions can occur in public facilities, such as at preschools, daycare centers attended after school, and in parks. In such places, if the scene of the accidental ingestion was not observed, it can often be difficult to determine the causative foreign body.

This study has highlighted two other interesting and important results. The number of patients with foreign-body ingestion differed by month; patients tended to present more frequently in the spring. In Japan, nursery, kindergarten, and school students begin their new school year during this period (the new semester starts in April). Due to a change in the living environment, such as when children move away from parental supervision, we speculate that the accidental ingestion of foreign bodies may increase during the spring. Secondly, while many children accidentally ingest foreign bodies [8,13], adult cases are usually due to psychiatric illness [14]. Some school-age patients have psychological or psychiatric disorders. In this current study, 50% of the six school-age patients who ingested internal medicines took an overdose. These cases were associated with children with psychological or psychiatric disorders. Schuldt et al. have reported that patients with psychological disorders and congenital malformations were high-risk patients for nasal and aural foreign-body ingestion [19]. When foreign-body ingestion occurs in school-age children, it is typically noticed and subsequently treated, especially when overdoses occur. Pediatricians and acute-care physicians should consider that school-age patients may have a background of psychological or psychiatric illness.

Our study does have limitations. Although a household’s economic status plays a role in the type of foreign body ingested, the data of a household’s economic status for each enrolled patient were not collected. This was solely a single-center retrospective cohort study, and in addition, the study field was a university hospital. Some patient selection bias may exist. However, this cohort’s clinical characteristics were not considerably different from those from the national database at the Ministry of Health, Labor and Welfare in Japan [20]. We posit that the results presented in this study are representative of Japan’s wider population. However, further large cohort studies utilizing national databases are required; these are important avenues of future research on this topic and may allow for a wider generalization of our results.

## 5. Conclusions

This is the first Japanese study to reveal that foreign bodies ingested by children differ according to age. In cases of unknown foreign-body ingestion, each age group should be specifically checked for certain foreign bodies. This information will be useful when examining pediatric patients who may have ingested a foreign body in a Japanese pediatric emergency or general practice setting.

## Figures and Tables

**Table 1:** Patient characteristics.

Background	N = 252
Age (years)	1 (0–15)
Male/Female	140 (55%)/112 (44%)
Recurrence	6 (2%)
Place of occurrence	
House	170 (67%)
Public accommodation	11 (4%)
Others	71 (28%)
First aid by bystanders	67 (27%)
**Symptoms at the time of ingestion**	
Absent	151 (60%)
Present	101 (40%)
Nausea and vomiting	39 (15%)
Cough	18 (7%)
Disturbance of consciousness	14 (5%)
Pharyngeal pain	9 (4%)
Displeasure	7 (3%)
Crying	6 (2%)
Abdominal pain and diarrhea	5 (2%)
Pale	2 (1%)
Respiratory distress	2 (1%)

Data are shown as median (range) or number (percent).

**Table 2:** Age-specific characteristics and major types of foreign bodies ingested.

Age	<12 Months *n* = 86	1 to 6 Years *n* = 146	≥7 Years *n* = 20	*p*-Value
Male/Female	48 (56%)/38 (44%)	82 (56%)/64 (44%)	10 (50%)/10 (50%)	0.872
Symptoms	38 (44%)	55 (38%)	8 (40%)	0.620
Occurrence at home	60 (70%)	101 (69%)	9 (45%)	0.082
First aid provided	30 (35%)	34 (23%)	3 (15%)	0.073
Major types				
Cigarettes	20 (23%)	23 (16%)	1 (5%)	<0.01
Plastics	27 (31%)	14 (10%)	2 (10%)	
Chemicals	8 (19%)	19 (13%)	0 (0%)	
Internal medicines	0 (0%)	20 (13%)	6 (30%)	
Metals	5 (6%)	18 (12%)	3 (15%)	
Others	26 (30%)	52 (36%)	8 (40%)	

Data are shown as median (range) or number (percent).
